# *GmBRC1* is a Candidate Gene for Branching in Soybean (*Glycine max* (L.) Merrill)

**DOI:** 10.3390/ijms20010135

**Published:** 2019-01-01

**Authors:** Sangrea Shim, Jungmin Ha, Moon Young Kim, Man Soo Choi, Sung-Taeg Kang, Soon-Chun Jeong, Jung-Kyung Moon, Suk-Ha Lee

**Affiliations:** 1 Department of Plant Science and Research Institute of Agriculture and Life Sciences, Seoul National University, Seoul 08826, Korea; sangreashim@gmail.com (S.S.); saga16@snu.ac.kr (J.H.); moonykim@snu.ac.kr (M.Y.K.); 2 Plant Genomics and Breeding Institute, Seoul National University, Seoul 08826, Korea; 3 National Institute of Crop Sciences, Rural Development Administration, Wanju-gun, Jeollabuk-do 55365, Korea; mschoi73@korea.kr; 4 Department of Crop Science & Biotechnology, Dankook University, Cheonan-si, Chungcheongnam-do 31116, Korea; kangst@dankook.ac.kr; 5 Bio-Evaluation Center, Korea Research Institute of Bioscience and Biotechnology, Cheongju-si, Chungcheongbuk-do 28116, Korea; scjeong@kribb.re.kr; 6 National Institute of Agricultural Sciences, Rural Development Administration, Jeonju-si, Jeollabuk-do 54874, Korea; moonjk2@korea.kr

**Keywords:** soybean, branching, genome-wide association study (GWAS), near-isogenic line (NIL), *BRANCHED1* (*BRC1*), TCP transcription factor

## Abstract

Branch number is one of the main factors affecting the yield of soybean (*Glycine max* (L.)). In this study, we conducted a genome-wide association study combined with linkage analysis for the identification of a candidate gene controlling soybean branching. Five quantitative trait nucleotides (QTNs) were associated with branch numbers in a soybean core collection. Among these QTNs, a linkage disequilibrium (LD) block *qtnBR6-1* spanning 20 genes was found to overlap a previously identified major quantitative trait locus *qBR6-1*. To validate and narrow down *qtnBR6-1*, we developed a set of near-isogenic lines (NILs) harboring high-branching (HB) and low-branching (LB) alleles of *qBR6-1*, with 99.96% isogenicity and different branch numbers. A cluster of single nucleotide polymorphisms (SNPs) segregating between NIL-HB and NIL-LB was located within the *qtnBR6-1* LD block. Among the five genes showing differential expression between NIL-HB and NIL-LB, *BRANCHED1* (*BRC1*; Glyma.06G210600) was down-regulated in the shoot apex of NIL-HB, and one missense mutation and two SNPs upstream of *BRC1* were associated with branch numbers in 59 additional soybean accessions. *BRC1* encodes TEOSINTE-BRANCHED1/CYCLOIDEA/PROLIFERATING CELL FACTORS 1 and 2 transcription factor and functions as a regulatory repressor of branching. On the basis of these results, we propose *BRC1* as a candidate gene for branching in soybean.

## 1. Introduction

Soybean (*Glycine max* (L.)) is a major food crop and a rich source of protein and oil in human diet and animal feed. Two cultivation methods with different planting densities are used to maximize soybean yield. The high-density planting method is mainly practiced in the USA [1], where the yield of soybean increases with planting density until saturation. The low-density planting method is practiced in Asia not only to avoid disease and lodging, but also to reduce seed and labor cost; however, the productivity in low-density planting is lower than that in high-density planting [2]. An important factor of low-density planting is branching plasticity, which offsets yield losses [3]. Branching pattern and branch number are dependent on environmental factors such as planting density and light quality [3,4,5]. These factors have obstructed the identification of genes regulating branch development. However, variation in branch number among soybean cultivars of diverse origins under low-density planting suggests the presence of genetic differences [6,7]. Thus, breeding for soybean genotypes with optimal plant architecture adapted to a specific planting density is necessary for improving yield and enabling mechanical harvesting.

To date, a dozen quantitative trait loci (QTLs) regulating branch development have been identified using recombinant inbred line (RIL) or F_2_ populations in soybean [7,8,9,10,11]. However, the identified QTLs span a large number of plausible genes because of the low resolution of genetic linkage maps and low recombination frequency in mapping populations. Recently, three known QTLs have been narrowed down to identify candidate genes based on a high-density linkage map using the 6K single nucleotide polymorphism (SNP) chip (BARCSoySNP6K), and positive phenotypic correlation has been demonstrated between branch number and total pod number [8]. Among the QTLs, a major one (*qBR6-1*) on chromosome (Chr) 6, with a logarithm of odds (LOD) score of 10.3 and 14.5% of the phenotypic variation in branch numbers, was shown to contain 13 genes [8]. One of these genes was a gene encoding TEOSINTE-BRANCHED1/CYCLOIDEA/PROLIFERATING CELL FACTORS 1 and 2 (TCP) transcription factor, also known as *BRANCHED1* (*BRC1*), which is involved in gene networks of axillary branching via interactions with the auxin hormone network [8]. In soybean, the *BRC1* gene has not been genetically identified.

A genome-wide association study (GWAS) is used to identify associations between genetic loci and traits. It is a powerful method, as it provides high genetic resolution derived from all recombination events that occurred during the evolution of a natural population [12]. GWAS has been used successfully to identify the genetic basis of complex agronomic traits in Arabidopsis (*Arabidopsis thaliana* (L.)), rice (*Oryza sativa* (L.)), and maize (*Zea mays* (L.)) [13,14,15]. In soybean, GWAS has been performed to identify the association of genetic regions with agronomic traits, including flowering time, maturity date, plant height, and seed oil content [16,17]. However, the detection of false-positive associations with phenotypes is a weakness of GWAS, which is caused by the population structure and kinship relatedness in natural populations [16]. Utilizing linkage analysis in combination with GWAS has been previously used to overcome the limitations of QTL mapping and to validate the results of GWAS [16].

The objective of this study was to narrow down a genomic region of *qBR6-1* to identify a candidate gene responsible for soybean branching. To this end, GWAS was conducted to identify quantitative trait nucleotides (QTNs) associated with branch number in a soybean core collection comprising 400 soybean genotypes grown in three locations. To validate and narrow down a QTN that was found to be overlapped with the previously identified major QTL, *qBR6-1*, we developed and analyzed a set of near-isogenic lines (NILs) carrying high-branching (HB) and low-branching (LB) alleles of *qBR6-1* derived from an F_6_ residual heterozygous line (RHL) heterozygous for *qBR6-1*. Using these NILs, we detected differential expression of genes within the linkage disequilibrium (LD) block of the QTN on Chr 6. In addition, we examined nucleotide variations in a selected candidate gene between NIL-HB and NIL-LB, and confirmed their allelic associations with branch numbers in soybean accessions obtained from the United States Department of Agriculture Germplasm Resources Information Network (USDA-GRIN). This study will provide a better understanding of genetic basis underlying the branch development in soybean and valuable information to improve plant architecture for soybean cultivars with high yields.

## 2. Results

### 2.1. Variation and Heritability in Branch Number of the Soybean Core Collection 

We present variations in branch number of the soybean core collection according to the location of cultivation (Wanju, Cheonan, and Ochang) and best linear unbiased predictor (BLUP) values in Figure S1. The number of branches varied from 2.0–20.7 in Wanju, 1.0–21.3 in Cheonan, and 0.0–14.3 in Ochang (Table S1). The broad-sense heritability (*H*^2^) of branch numbers in the soybean core collection was 57.7% (Table S1). Analysis of variance (ANOVA) showed significant effects of genotype as well as genotype-by-environment (G × E) interaction on branch numbers (*p* < 0.0001). Additionally, a continuous distribution of branch numbers was observed in all three locations, indicating that branch numbers were regulated by multiple genetic factors. To identify reliable QTNs associated with branch numbers, the BLUP values were calculated for each genotype and used in GWAS (Figure S1). 

### 2.2. Population Structure and LD

**Figure 1:**
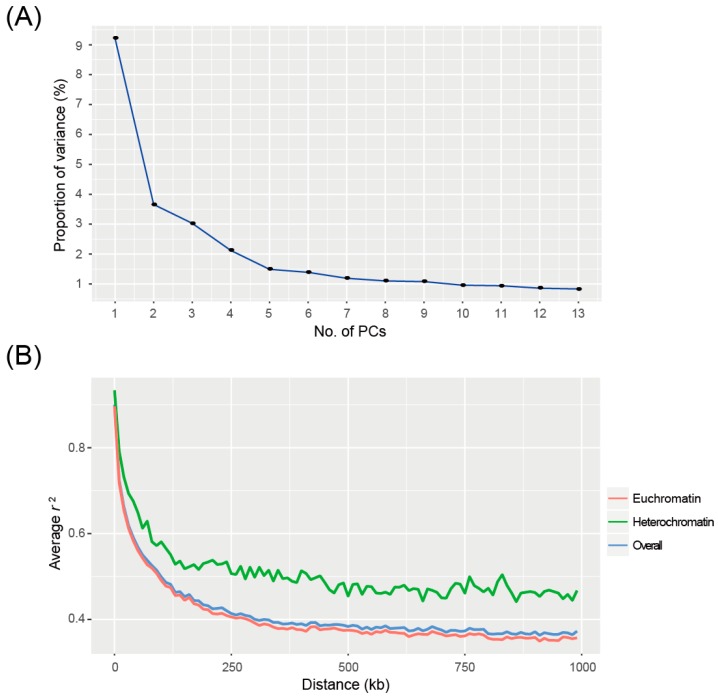
Principal components analysis (PCA) and linkage disequilibrium (LD) decay of the soybean core collection. (**A**) Scree plot showing the proportion of variance explained by principal components (PCs). (**B**) LD decay based on pairwise *r*^2^ values. Blue line represents the overall rate of LD decay. Red and green lines indicate LD decay patterns of euchromatin and heterochromatin, respectively.

Covariates from the population structure were stratified using 81,078 SNP markers. The log likelihood (LnP(D)) from STRUCTURE [18] analysis showed a continuous increase with the number of sub-populations (*K*). Therefore, we determined the optimum *K* value using the ∆*K* method. The ∆*K* value classified the core collection into two groups (Figure S2). In a scree plot, the proportion of variance drastically decreased until the number of principal components (PCs) reached two (Figure 1A). The extent of LD estimated by PLINK [19] showed that the average pair-wise squared correlation coefficient (*r*^2^) between alleles was dropped to half of its maximum value at 160–170 kb for the entire genome, with 140–150 kb for euchromatin and 460–470 kb for heterochromatin (Figure 1B). 

### 2.3. Determination of Genetic Association with Branch Numbers Using GWAS

To determine the best fitted GWAS model for branch numbers, quantile–quantile (QQ) plots generated using the generalized linear model (GLM) + population structure (Q) were compared with those generated using the mixed linear model (MLM) + Q (from principal component analysis; PCA) and kinship matrix (K) (Figure S3). The GLM + Q model showed a strong inflation of *p*-value (blue dots) compared with the MLM + Q (PCA) and K model (red dots), indicating erroneous inflation of false-positive signal. Thus, MLM + Q (PCA) and K model was more appropriate for the identification of QTNs associated with branch numbers in this study.

**Figure 2:**
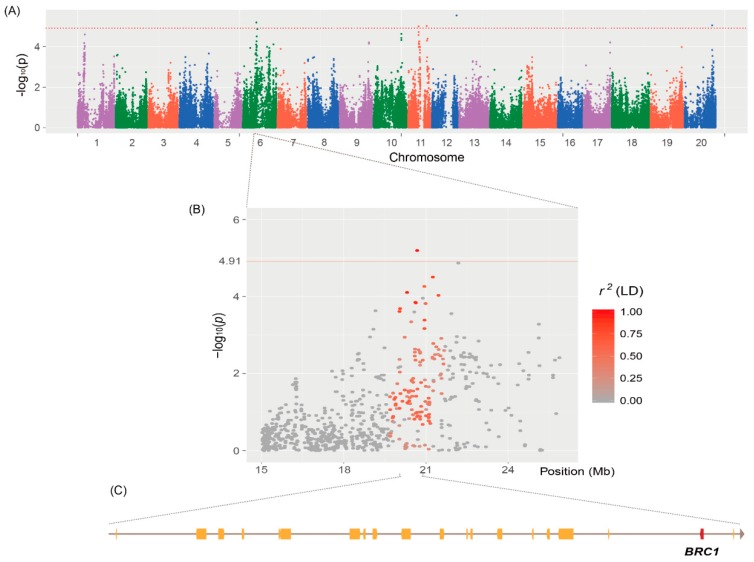
Genome-wide association study (GWAS) of branch numbers in the soybean core collection. (**A**) Genome-wide Manhattan plot of branch numbers. (**B**) LD region harboring *qtnBR6-1* on Chr 6. The pair-wise *r*^2^ values between markers in LD are presented along a color gradient ranging from gray to red. The *p*-value threshold is indicated with red horizontal lines in Manhattan plots. (**C**) Protein-coding genes located in the LD block of *qtnBR6-1*.

A total of five significant QTNs showing significant association with branch numbers were identified on Chr 6, 11, 12, and 20 (Table 1 and Figure 2A). The most highly significant QTN, *qtnBR12-1* at 38,057,780 base pair (bp) in the euchromatic region of Chr 12, explained 6.4% of the phenotypic variation in branch numbers. The second most highly significant QTN, *qtnBR6-1* at 20,663,101 bp in heterochromatin of Chr 6, accounted for 5.8% of the phenotypic variation (Figure 2B). Phenotypic variation in branch numbers explained by two other QTNs, *qtnBR11-1* and *qtnBR11-2* at 16,074,992 and 28,613,118 bp, respectively, in heterochromatin of Chr 11 was 5.0% and 5.6%, respectively. The last QTN, *qtnBR20-1*, located at 42,471,316 bp in euchromatin of Chr 20, accounted for 4.9% of the phenotypic variation in branch numbers. 

**Figure 3:**
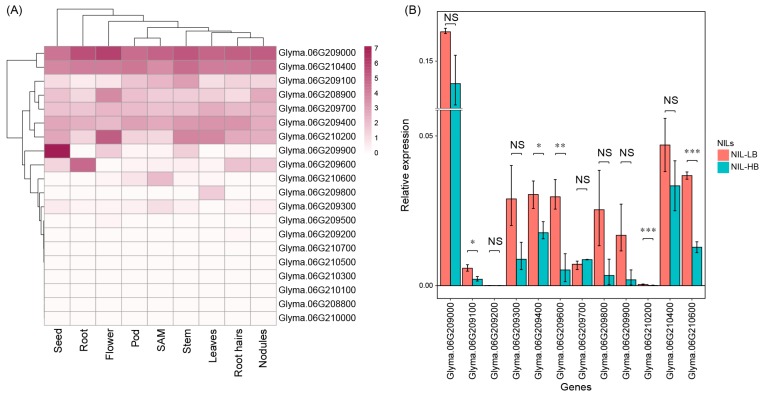
Expression patterns of genes located in the LD block of *qtnBR6-1*. (**A**) Heatmap showing the expression patterns of 20 genes obtained from the publicly available RNA-seq data. Gene expression values of log_2_(FPKM+1) were used to generate the heatmap. (**B**) Comparison of expression levels of selected genes in the shoot apical meristem between NILs with high-branching (HB) and low-branching (LB) alleles (NIL-HB and NIL-LB, respectively). The red and green bars indicate mean expression level of genes for nine samples (three biological replicates x three technical replicates) of each NIL (NIL-LB and NIL-HB), respectively. Black variance bars represent standard error of the mean (SEM). Statistically significant differences in relative gene expression between NIL-HB and NIL-LB are indicated with asterisks (*, *p* < 0.05; **, *p* < 0.01; ***, *p* < 0.001). NS, not significant.

On the basis of the rate of LD decay, we extended the chromosomal regions both upstream and downstream of the QTN positions, up to 140 kb for euchromatin and 460 kb for heterochromatin (Table 1). The LD blocks of *qtnBR6-1*, *qtnBR11-1*, and *qtnBR11-2* were adjacent to a major QTL *qBR6-1* and a minor QTL *qBR11-1* reported previously [8]. The *qtnBR6-1*, spanning 20 protein-coding genes (Figure 2C), overlapped with the QTL *qBR6-1*, which has been shown to play a major role in branch development. Using the publicly available soybean RNA-seq data [20], in silico expression profiling of all 20 genes in *qtnBR6-1* revealed that 13 genes, including the TCP transcription factor gene (*BRC1*; Glyma.06G210600), were expressed in the shoot apical meristem (SAM) (Figure 3A). 

### 2.4. Isogenicity and Phenotypic Differences Between NIL-HB and NIL-LB Associated with qBR6-1

**Figure 4:**
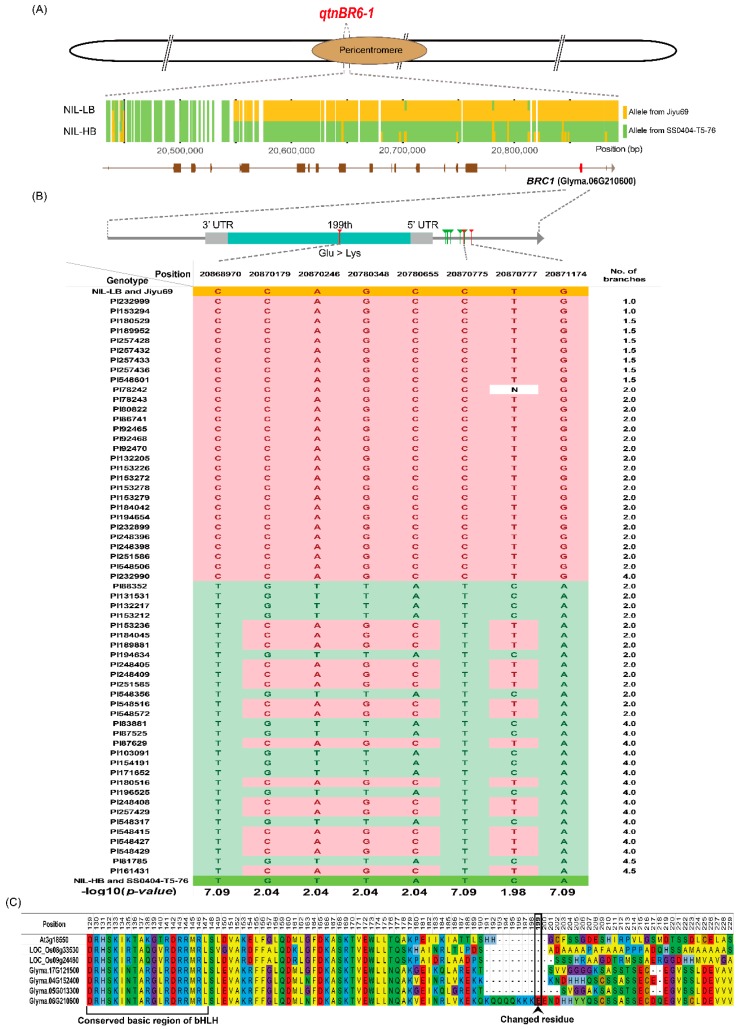
Allelic association of nucleotide variants in *BRC1* with branch numbers and protein sequence alignment of *BRC1*. (**A**) Physical location of *qtnBR6-1* in the pericentromeric region of Chr 6, and a cluster of single nucleotide polymorphism (SNPs) segregating between NIL-HB and NIL-LB. The SNPs between NIL-HB and NIL-LB within the LD block of *qtnBR6-1* are originated from SS0404-T5-76 (high-branching, green) and Jiyu69 (low-branching, orange), respectively. The X-axis indicates the genomic position (bp). (**B**) Allelic association of SNPs in *BRC1* with branch numbers among 59 United States Department of Agriculture (USDA) soybean accessions. Among eight SNPs, one missense mutation and two SNPs upstream of *BRC1*, indicated by red bars, were associated with branch numbers in 59 USDA soybean accessions. (**C**) Protein sequence alignment of *BRC1* orthologues. The missense mutation (glutamate to lysine) at amino acid position 199 is outlined with a black box.

To validate and narrow down *qtnBR6-1*, we explored a set of NILs carrying HB and LB alleles of *qBR6-1*. NIL-HB with more branches contained HB allele derived from paternal genotype SS0404-T5-76. NIL-LB carried LB allele from maternal genotype Jiyu69, showing fewer branches. The NILs and parental genotypes, SS0404-T5-76 and Jilyu69, were resequenced at an average depth of 31.6X (Table S2). On average, 92.4% of the paired-end reads were mapped to the soybean reference genome sequence. Within 895 Mb of consensus genome sequence of the NILs with at least 10X mapping depth, a total of 286,467 nucleotide variants were identified on all chromosomes except Chr 6 (carrier chromosome) between NIL-HB and NIL-LB, resulting in 99.97% isogenicity (Table S3). In addition, the non-QTL region on the carrier Chr 6 showed 99.89% isogenicity. The QTL region of *qBR6-1* on Chr 6 harbored 3798 nucleotide variants between NIL-HB and NIL-LB, of which 96.2% were shared with polymorphisms between parental genotypes SS0404-T5-76 and Jilyu69. Within the LD block of *qtnBR6-1*, SNPs segregating between NIL-HB and NIL-LB clustered in a 343 kb interval (20,555–20,898 kb) harboring 16 protein-coding genes (Figure 4A). 

Additionally, we evaluated the effect of planting density on the branching performance of NIL-HB and NIL-LB in the field and greenhouse. The results showed a significant difference in the number of branches between NIL-HB and NIL-LB grown under low planting density (Table 2 and Figure S4). On average, NIL-HB plants had two more branches than NIL-LB plants. High planting density displayed no difference in branch numbers between NIL-HB and NIL-LB.

### 2.5. Candidate Gene Identification and Allelic Association Analysis 

To narrow down the 13 candidate genes identified in the LD block of *qtnBR6-1*, cDNA was isolated from the shoot apex of NILs and used for expression analysis by qRT-PCR. Among these 13 genes, a multi-copy gene (Glyma.06G208900) encoding ATPase E1-E2 type family protein could not be analyzed by qRT-PCR because the primers were not sequence-specific. Of the remaining 12 genes, five were significantly down-regulated in NIL-HB; the genes encode MIZU-KUSSEI-like protein (Glyma.06G209100), P-loop containing nucleoside triphosphate hydrolases superfamily protein (Glyma.06G209400), adenine nucleotide alpha hydrolases-like superfamily protein (Glyma.06G209600), unknown protein (Glyma.06G210200), and TCP transcription factor (*BRC1*; Glyma.06G210600) (Figure 3B). On the basis of molecular genetic data available in Arabidopsis [21,22], *BRC1* was identified as the most promising candidate gene responsible for branch development in soybean. 

Analysis of the nucleotide sequence of *BRC1* in NILs, Jily69, and SS0404-T5-76 revealed one SNP in the coding sequence, resulting in a missense mutation at amino acid position 199 (glutamate to lysine), and seven additional SNPs in the 2 kb upstream sequence (Figure 4B). Furthermore, the SNP in *BRC1* coding sequence and two SNPs in the upstream sequence were significantly associated with branch number in 59 USDA-GRIN soybean accessions (available online: http://www.ars-grin.gov).

## 3. Discussion

Shoot branching influences seed yield in soybean and is regulated by a complex mechanism of axillary bud outgrowth following axillary meristem initiation [21,23]. The fate of axillary buds, that is, whether to outgrow into a branch or to remain as a bud, is determined by an orchestrated regulatory process induced by endogenous hormonal and developmental signals [24]. Such a regulatory process is affected by environmental factors such as planting density, shading, light quality, soil nitrogen content, and soil water content [3,4,25,26,27]. The soybean core collection used in this study showed a more significant effect on branch numbers than locations or interactions between genotypes and locations. We identified five QTNs showing association with branch numbers based on BLUP values from three different locations (Table 1 and Figure 2). The LD block of one of these five QTNs, *qtnBR6-1*, co-localized with a previously reported major QTL, *qBR6-1*, which was validated based on the clustering of SNPs segregating between NIL-HB and NIL-LB (Figure 4A).

The outgrowth of axillary buds is inhibited by the active shoot apex; this phenomenon is referred to as apical dominance. Decapitation abolishes apical dominance and triggers the growth of one or more axillary buds because auxin, which is synthesized in the shoot apex, is mobilized to the lower parts of plants and inhibits branch outgrowth [28]. Considering the relevance of shoot apex to branch development, we compared the expression levels of 13 genes showing transcriptional activity in SAM using publicly available RNA-seq data (Figure 3A). Among the five significantly down-regulated genes in NIL-HB (Figure 3B), we identified *BRC1* (Glyma.06G210600) as the most promising candidate gene responsible for soybean shoot branching because *BRC1* functions as a regulatory hub that integrates hormonal signals and external stimuli for determining the fate of axillary buds [21,22]. In Arabidopsis, *BRC1* is expressed in axillary buds and SAM [21,29]. In pea (*Pisum sativum* L.), *PsBRC1* shows the highest expression level in lateral buds and is also expressed in the shoot apex [30]. In this study, NIL-LB showed higher transcriptional activity of *BRC1* in the shoot apex than NIL-HB, although the expression of *BRC1* was not examined in axillary buds. Our data are consistent with previous studies showing that *BRC1* orthologues in rice (*Ostb1*) and maize (*tb1*) negatively regulate branch development [21,22,31,32]. 

The effects of planting density and shading on branch development are well-established in plants [3,5,21,33,34]. High planting density and shading have the same effect on light quality as they both reduce the ratio of red to far red light (R/FR) [24]. Shoot branching increases under high R/FR ratios caused by low planting density, but decreases under low R/FR ratios [3,5,21,33,34]. Similarly, in this study, NILs grown under high planting density produced fewer branches than those grown under low planting density, and showed no difference in branch numbers (Table 2). However, a significant difference in branch numbers was observed between NIL-HB and NIL-LB cultivated at low planting density. This indicates that the gene regulating branch number responds to low planting density. The light signal perceived by phytochrome B (PHYB), a photoreceptor, is transduced to the endogenous signal via *BRC1*, which functions as a molecular mediator [34]. Under shade or high planting density, an elevated FR signal inactivates PHYB by converting the active form of phytochrome (Pr) to the inactive form (Pfr) [35]. The inactive Pfr form of PHYB up-regulates *BRC1*, resulting in the inhibition of branch development [34,35]. These findings link the exogenous light signal with the endogenous molecular regulator in branch development, and suggest *GmBRC1* as the causal gene responsible for branch development in soybean. The function of *BRC1* is highly conserved across other plant species [21,30,31,32]. 

The *BRC1* gene and its orthologues are characterized by a highly conserved basic region of basic helix–loop–helix (bHLH) on a functional domain of TCP genes [21,31,32,36], and belong to CYCLODEA/TEOSINTE BRANCHED1 (CYC/TB1)-type TCP [37]. In this study, a missense mutation (glutamate to lysine) in the CYC/TB1-type TCP domain was identified between NIL-HB and NIL-LB, which showed a tight association with branch numbers in 59 USDA soybean accessions (Figure 4B). However, the altered amino acid residue was not located in the highly conserved basic region of bHLH, and was not conserved among other CYC/TB1-type TCP orthologues in Arabidopsis, rice, and soybean (Figure 4C). Thus, it is not clear if the difference in branch numbers between NIL-HB and NIL-LB could be attributed to the amino acid change at position 199. In addition to the SNP in *BRC1* coding sequence, two out of seven SNPs within the 2 kb upstream sequence of *BRC1* showed tight association with branch numbers. As both these SNPs are located in the putative promoter region of *BRC1*, they are predicted to affect *BRC1* expression. This is consistent with previous reports that *BRC1* regulates branching at the transcription level [21,31,32]; *BRC1* expression was down-regulated in NIL-HB (Figure 3B). An example similar to our results is of maize *tb1*; sequence variation in the upstream region of *tb1*, resulting in low expression, is associated with increased branch development in maize [31,38,39].

In conclusion, we propose *BRC1* (Glyma.06G210600) as the candidate gene regulating branch development in soybean. Further functional validation of these results by overexpression or knockout of *GmBRC1* is required for a thorough understanding of the regulatory mechanism of branch development in soybean, which will provide key insights into the complex genetic modules mediating branch development in soybean. Agronomically, soybean cultivars with optimal plant architecture depending on cultivation methods can be developed based on the allelic information of *BRC1* gene. In western countries including USA, soybean cultivars for high yield and mechanical harvesting can be improved by selecting genotypes with alleles contributing low branching phenotype. Besides, introgression of alleles responsible for high branching phenotype to other elite cultivars will enable breeding of high yielding soybean cultivars with high branch number and contribute to labor saving cultivation practice in Asian countries.

## 4. Materials and Methods

### 4.1. Plant Materials

A soybean core collection comprising 400 soybean genotypes with diverse origins was obtained from the National Agrobiodiversity Center in the Rural Development Administration (RDA, Jeonju, Korea) for GWAS (Table S4). To validate and narrow down the locus for branching, a set of NILs carrying HB and LB alleles of *qBR6-1* was developed from an RHL selected from the F_6_ RIL population of Jiyu69 (low-branching) × SS0404-T5-76 (high-branching). Among five individuals in the progeny of RHL, plants showing the highest and lowest number of branches were selected as NILs carrying the HB and LB alleles, respectively, in 2016, and were designated as NIL-HB and NIL-LB, respectively. These NILs were genotyped using simple sequence repeat markers flanking *qBR6-1* (Table S5). We also used 59 USDA-GRIN soybean accessions with known branch numbers to confirm the allelic association of SNPs within a candidate gene between NIL-HB and NIL-LB. 

The soybean core collection was grown in three different locations, namely, Wanju (35°50′27.384″ N, 127°2′46.1826″ E), Cheonan (36°49′49.2816″ N, 127°10′1.9122″ E), and Ochang (36°43′14.0982″ N, 127°26′1.1148″ E), in Korea in 2017. NIL-HB and NIL-LB were planted in a greenhouse and experimental field of Seoul National University, Suwon, Korea (37°16′12.094″ N, 126°59′20.756″ E). In the greenhouse, three plants of each line (NIL-HB and NIL-LB) were grown in a rectangular pot (64.3 cm × 23.0 cm × 16.9 cm). The plant-to-plant and row-to-row spacing was 20 and 80 cm, respectively, in low-density planting, and 10 and 40 cm, respectively, in high-density planting. All experiments were performed in triplicate, and field evaluation of branching in NILs was conducted in 2017 and 2018.

### 4.2. Phenotyping of Branch Number and Statistical Analysis

The number of branches generated on the main stem of the soybean core collection genotypes and NILs was evaluated in three biological replicates. Phenotypic differences between NILs were examined by analysis of variance (ANOVA) using the R software (available online: http://www.R-project.org). To minimize the effect of environmental factors on branch numbers at three different locations in GWAS, the BLUP value was predicted by the lme4 package of R, considering the variation among genotypes and locations [40]. The BLUP values were calculated according to the following equation:

$$Y_{ik}=\mu +G_{i}+L_{k}+GL_{ik}+e_{ik}$$

where *Y_ik_* represents the phenotypic measurement, *μ* is the total mean, *G_i_* is the genotypic effect of the *i^th^* genotype, *L_k_* is the effect of the *k^th^* location, *GL_ik_* represents interaction between genotype and location, and *e_ik_* is the residual error. The BLUP values of each soybean genotype were calculated with random effect and used as phenotypes for GWAS. Broad-sense heritability (*H*^2^) of branch numbers was calculated using the following equation:

$$H^{2}=\frac{\sigma_{g}^{2}}{\left(\sigma_{g}^{2}+\frac{\sigma_{gl}^{2}}{n}+\frac{\sigma_{e}^{2}}{nr}\right)}$$

where *σ_g_*^2^ represents genotypic variance, *σ_gi_*^2^ is the variance of interaction between genotype and location, *σ_e_*^2^ represents the variance of error components, *n* represents the number of locations, and *r* represents the number of replications.

### 4.3. Population Structure and LD Analysis

SNP genotypic data of the soybean core collection previously produced from the 180K Axiom^®^ SoyaSNP array were explored for GWAS [41]. SNPs with minor allele frequency <0.05 and missing genotype >10% were excluded. The remaining 81,078 SNPs were used in STRUCTURE [18], PCA, kinship analysis, LD analysis, and GWAS. To stratify covariates (Q) from the population structure, STRUCTURE and PCA analyses were applied. In STRUCTURE, burn-in and Markov chain Monte Carlo (MCMC) values were set at 10,000 and 100,000, respectively. The STRUCTURE analysis was carried out for number of sub-populations (*K*) values ranging from 1 to 13. To evaluate the optimum *K* for this population, the ∆*K* method was applied. The use of MLM with covariates from STRUCTURE produced erroneous inflation and false-positive signals. Therefore, population stratification was analyzed using PCA, which has been previously proposed as an alternative method for investigating relatedness [42]. Therefore, covariates implemented by PCA analysis in TASSEL v5.2 [43] were adopted for MLM analysis. To determine the optimal number of PCs, a scree plot was generated based on the proportion of variance explained by PCs. The kinship matrix (K) for soybean core collection was analyzed using TASSEL v5.2 [43]. 

LD was analyzed using PLINK software [19] with LD window length of 1 Mb and an unlimited number of variants within LD window (--r2 --ld-window-kb 1000 --ld-window 99999). Considering the different patterns of LD decay in heterochromatin and euchromatin, genomic regions specified as pericentromeric regions in Soybase (available online: http://soybase.org) were downloaded and used in the LD analysis. The rate of LD decay for the soybean core collection was measured in physical distance, where the average pair-wise *r*^2^ value between alleles dropped to half of its maximum value.

### 4.4. GWAS

GWAS was conducted using TASSEL v5.2 [43]. Two statistical models, GLM + Q (from STRUCTURE) and MLM + Q (from PCA) and K, were considered. The Q and K were regarded as fixed and random effects in GLM and MLM models, respectively. Quantile–quantile plots of both models were compared for determining the best fit. The threshold *p*-value (1/*n*, where *n* is the number of SNPs (81,078)) was used for the identification of QTNs significantly associated with branch number.

### 4.5. Expression Patterns of Genes in LD Block of qtnBR6-1

The identified QTNs were extended based on the rate of LD decay, depending on the chromosomal region (euchromatin vs. heterochromatin). To investigate the expression patterns and levels of genes located within the LD block of the *qtnBR6-1*, RNA-seq data (fragments per kilobase of exon model per million mapped reads (FPKM) values) for nine tissues, including flower, leaf, nodule, pod, root, root hair, SAM, seed, and stem, of soybean cv. Williams 82 were obtained from Phytozome v12.0 (available online: https://phytozome.jgi.doe.gov/pz/portal.html) [20]; RNA-seq data for axillary buds were not available in the public database. A heatmap with hierarchical clustering of genes was constructed using the R package pheatmap for visualizing gene expression levels in nine tissues, based on the log_2_(FPKM + 1) values. Only genes expressed in SAM were selected for further analysis. 

### 4.6. Resequencing of NIL-HB, NIL-LB, and Parental Genotypes

The two NILs (NIL-HB and NIL-LB) and their parental genotypes, SS0404-T5-76 and Jiyu69, were resequenced. Raw sequence reads were mapped to the soybean reference genome (Wm82.a2) downloaded from Phytozome [20] using BWA [44], Samtools [45], and Vcftools [46]. Annotation of SNPs was conducted using SnpEff [47]. Nucleotide positions with more than ten supporting reads per genotype were analyzed further. SNPs segregating between NIL-HB and NIL-LB in the LD block of *qtnBR6-1* were compared with the sequence of SS0404-T5-76 and Jiyu69.

### 4.7. qRT-PCR Analysis of Candidate Genes in NILs

To determine the expression levels of selected genes in NILs, total RNA was extracted from the shoot apex (<3 mm) of NILs at the R1 stage using Ribospin^TM^ Plant (GeneAll, Seoul, Korea), and cDNA was synthesized using Bio-Rad iScript^TM^ cDNA Synthesis Kit (Hercules, CA, USA). Next, qRT-PCR was performed on a LightCycler^®^ 480 (Roche Diagnostics, Laval, QC, Canada) using Bio-Rad iQ^TM^ SYBR Green Supermix Kit. Primer sequences were designed using PRIMER3plus (available online: http://www.bioinformatics.nl/cgi-bin/primer3plus/primer3plus.cgi) [48]. Appropriate primer pairs that did not amplify orthologues of target genes were selected using a stand-alone version of electronic PCR [49] (Table S5). Each qRT-PCR reaction mixture (20 µL volume) contained 100 ng cDNA template, and 300 µM each of forward and reverse primer. Amplification was performed using the following conditions: initial denaturation at 95 °C for 5 min, followed by 40 cycles of denaturation at 95 °C for 10 s, and annealing and extension at 60 °C for 1 min. Three biological samples of each NIL were analyzed in triplicate to increase statistical power. The *ACTIN11* (*ACT11*) gene was used as a reference for data normalization. Normalized data were analyzed using the method of Livak and Schmittgen [50]. Statistical significance was analyzed using Fisher’s least significant difference (*p* < 0.05) in R.

### 4.8. Analysis of BRC1 SNPs and Amino Acid Sequence

SNPs identified in the candidate gene *BRC1* based on a comparison between NIL-HB and NIL-LB were tested for association with branch number in a collection of 59 soybean accessions obtained from USDA-GRIN. Genomic DNA of each soybean accession was extracted using Exgene^TM^ Plant SV mini DNA extraction kit (GeneAll, Seoul, Korea). On the basis of the identified SNPs between NIL-HB and NIL-LB, primers were designed using PRIMER3Plus (Table S5) [48] and validated using an electronic PCR algorithm [49]. PCR products were sequenced using ABI 3730XL DNA analyzer (Applied Biosystems, Foster, CA, USA). Branch numbers of soybean accessions were downloaded from the GRIN website. Association analysis between branch numbers and allelic variations was performed using ANOVA. Amino acid sequences of *BRC1* orthologues from Arabidopsis, rice, and soybean were aligned using MEGA7 [51].

## Figures and Tables

**Table 1:** Quantitative trait nucleotides (QTNs) associated with branch numbers in the soybean core collection identified by genome-wide association study (GWAS).

QTN ID	Marker ID	Chr ^a^	Marker Position (bp)	*p*-Value	Phenotypic R^2^ (%)	Chromosomal Location	Linkage Disequilibrium Block		No. of Genes	Known QTL	Reference ^b^
*qtnBR6-1*	AX-90305605	6	20,663,101	6.43 × 10^−6^	5.8	Heterochromatin	20,433,101	20,893,101	20	*qBR6-1*	[8]
*qtnBR11-1*	AX-90512426	11	16,074,992	9.98 × 10^−6^	5.0	Heterochromatin	15,844,992	16,304,992	13	*qBR11-1*	[8]
*qtnBR11-2*	AX-90472718	11	28,613,118	9.51 × 10^−6^	5.6	Heterochromatin	28,383,118	28,843,118	23	*qBR11-1*	[8]
*qtnBR12-1*	AX-90419363	12	38,057,780	2.89 × 10^−6^	6.4	Euchromatin	37,987,780	38,127,780	14		
*qtnBR20-1*	AX-90519199	20	42,471,316	8.88 × 10^−6^	4.9	Euchromatin	42,401,316	42,541,316	13		

^a^ Chr represents the soybean chromosome. ^b^ Reference represented reference article for previously reported quantitative trait loci (QTL).

**Table 2:** Effect of planting density on the branch number of soybean near-isogenic lines (NILs) carrying high-branching (HB) and low-branching (LB) alleles (NIL-HB and NIL-LB, respectively) at the quantitative trait loci (QTL) *qBR6-1*.

Year	Planting Density	Growth Condition	NIL-HB	NIL-LB	*p*-Value
2017	Low	Greenhouse	5.8 ± 1.7	3.4 ± 0.7	0.0001
		Field	14.5 ± 1.1	12.2 ± 2.1	0.019
2018	Low	Field	7.1 ± 1.4	5.1 ± 1.6	0.0003
	High		3.3 ± 1.9	2.6 ± 0.8	0.057

## Supplementary Materials

Supplementary materials can be found at http://www.mdpi.com/1422-0067/20/1/135/s1.

## Abbreviations

BLUP	Best linear unbiased predictor/prediction
*BRC1*	*BRANCHED1*
GLM	Generalized linear model
GWAS	Genome-wide association study
LD	Linkage disequilibrium
MLM	Mixed linear model
NIL	Near-isogenic line
QTL	Quantitative trait locus
QTN	Quantitative trait nucleotide
RHL	Residual heterozygous line
RIL	Recombinant inbred line
SAM	Shoot apical meristem
SNP	Single nucleotide polymorphism
TCP	TEOSINTE BRANCHED1/CYCLODEA/PROLIFERATING CELL FACTOR 1 and 2
