# Supplementary material for: GmBRC1 is a Candidate Gene for Branching in Soybean (Glycine max (L.) Merrill)
Source: Int J Mol Sci. 2019 Jan 1;20(1):135. doi: 10.3390/ijms20010135 (PMC6337253; doi:10.3390/ijms20010135)
Supplement: Supplementary file 1 [file ijms-20-00135-s001.pdf]

Table S1: Statistical analysis and broad-sense heritability of branch numbers in the soybean core collection comprising 400 genotypes grown in three different locations.

Location [a]	Branch numbers		F_G [c]	$F_{G \times E}$ [c]	H^2 (%) [d]
	Mean ± SD [b]	Range			
Wanju	7.5 ± 2.3	2.0 – 20.7	6.9***	3.3***	57.7
Cheonan	6.8 ± 2.7	1.0 – 21.3			
Ochang	6.2 ± 2.7	0.0 – 14.3			

[a] Location represents locations of branch number measured.

[b] Mean ± SD indicate mean value and standard deviation of phenotype measurement.

[c] F_G and $F_{G \times E}$ represent *F-value* of genotypic and interaction of genotype and environmental effects.

[d] H^2 (%) represent broad-sense heritability in percent.

Table S2: Mapping statistics of NGS reads from parental lines and NILs.

	Jiyu69(ratio)		SS0404-T5-76(ratio)		NIL-LB(ratio)		NIL-HB(ratio)	
No. of total reads	240,197,070	(N/A)	199,066,733	(N/A)	235,624,767	(N/A)	219,702,872	(N/A)
No. of mapped reads	238,925,979	(99.47%)	197,970,721	(99.45%)	234,145,171	(99.37%)	218,630,871	(99.51%)
No. of mapped reads (paired)	216,769,298	(91.19%)	182,554,210	(92.48%)	212,600,360	(91.13%)	206,800,718	(94.70%)
No. of singltons	673,027	(0.28%)	563,104	(0.29%)	701,226	(0.30%)	532,309	(0.24%)
Average depth	33.45	(N/A)	28.17	(N/A)	32.81	(N/A)	31.91	(N/A)

Table S3: Statistics of background selection for NILs.

Consensus length	Variant type	Chromosomes excepting Chr. 6			Carrier chromosome (Chr. 6)						Isogenisity [a] (Overall)
					Non-QTL			QTL			
		Monomorphic	Polymorphic	Isogenisity [a]	Monomorphic	Polymorphic	Isogenisity [a]	Monomorphic	Polymorphic	Shared [b]	
894,814,168	SNP	845,733,747	251,145	99.97	46,733,819	45,511	99.90	430,703	3,365	3,241	99.97
	INDEL	1,492,909	35,322	97.69	81,557	5,282	93.92	375	433	412	97.49
	Total	847,226,656	286,467	99.97	46,384,298	50,793	99.89	431,078	3,798	3,653	99.96

[a] Isogenisity was expressed in %.

[b] Shared represented number of polymorphic variants shared genotypes with parents.

Table S4: List of germplasms composing soybean core collection.

Core SET ID	Genotype name/ accession ID	Origin	Core SET ID	Genotype name/ accession ID	Origin
CC2-001	Geomeun	Korea, South	CC2-217	Ajuggali	Korea, South
CC2-002	Kyoungbuk Kyoungsan-1997-54	Korea, South	CC2-218	Suwongaetong #2	Korea, South
CC2-003	Jeonnam heanam-1998-16	Korea, South	CC2-220	PI 82218	Korea, South
CC2-004	PI 82278	Korea, South	CC2-221	IT177513	Korea, South
CC2-005	PI 399122	Korea, South	CC2-222	Ol	Korea, South
CC2-006	Wu tong shu huang dou	China	CC2-223	PI 458269	Korea, South
CC2-007	SLS90-101	Unknown	CC2-224	Kangwon sujib3-31	Korea, South
CC2-008	KLS86083	Korea, South	CC2-225	PI 91083	Korea, South
CC2-009	Tong xu xiao zi huang	China	CC2-226	PI 458175 C	Korea, South
CC2-010	PI 339736	Korea, South	CC2-227	KLS419	Korea, South
CC2-011	KLS88035	Korea, South	CC2-228	PI 84680	Korea, South
CC2-013	Nan zhao cao huang dou	China	CC2-229	Sochung2	Korea, South
CC2-014	PI 68696	China	CC2-230	Lindarin #63	USA
CC2-015	Baekjung #42	Korea, South	CC2-231	PI 80470	Japan
CC2-016	PI 90763	China	CC2-232	SS0404-T5-76	Korea, South
CC2-017	KAS 160-5	Korea, South	CC2-233	OT94-51	Canada
CC2-018	PI 86490	Japan	CC2-234	WIR2962	Korea, North
CC2-019	PI 82183	Korea, South	CC2-235	IT162760	Korea, South
CC2-021	Cai dou	China	CC2-236	KLS87113	Korea, South
CC2-022	KAS331-13	Korea, South	CC2-237	L-B	Korea, South
CC2-023	Keunol	Korea, South	CC2-238	SLSB-B-15	Korea, South
CC2-024	VIR 2977	Korea, North	CC2-239	Galchae	Korea, South
CC2-025	PI 84611	Korea, South	CC2-240	VIR 2978	Korea, North
CC2-026	IT103340	Korea, South	CC2-242	PI 96983	Korea, North
CC2-027	You huang dou	China	CC2-243	Tong shan huang da dou jia	China
CC2-028	Heugseagyuwoldu	Korea, South	CC2-244	KLS77013	Korea, South
CC2-029	IT178037	Korea, South	CC2-245	SLS B30-2	Taiwan
CC2-030	KAS361-2	Korea, South	CC2-246	PI 86982	Korea, South
CC2-031	Niu mao huang	China	CC2-247	Clark	USA
CC2-032	Bancheongdu	Korea, South	CC2-248	PI 84669 N	Korea, South
CC2-034	Ryumuo	Korea, North	CC2-249	York	USA
CC2-035	Mote	Korea, North	CC2-250	IT177518	Korea, South
CC2-036	KwangkyoV2	Korea, South	CC2-251	KAS651-37	Korea, South
CC2-037	KAS640-46	Korea, South	CC2-252	Lindou 9	Unknown
CC2-038	Suwon98	Korea, South	CC2-253	Sugae #43(A)	Korea, South
CC2-039	KLS85109	Korea, South	CC2-254	IT112859	Korea, South
CC2-040	Kangwon sujib2-33	Korea, South	CC2-255	CS 01964	Korea, South
CC2-041	KLG 16001	Korea, North	CC2-256	KLS77131-2	Korea, South
CC2-042	Kwangan	Korea, South	CC2-257	KLS087160	Korea, South
CC2-043	KLS87277	Korea, South	CC2-258	Hannam	Korea, South
CC2-044	CS 02005	Korea, South	CC2-259	KAS 100-12	Korea, South
CC2-045	SLSB397-1	Korea, South	CC2-260	PI 87630	Japan
CC2-046	Bosug	Korea, South	CC2-261	Yugwu16	Japan
CC2-047	PI 407736	China	CC2-262	Hwangkeum	Korea, South
CC2-048	PI 475824 A	China	CC2-263	PI 96280	Korea, North
CC2-049	KLS72O-1	Korea, South	CC2-264	Nonduleong	Korea, South
CC2-050	KAS302-19	Korea, South	CC2-265	Daechu	Korea, South
CC2-051	PI 84609	Korea, South	CC2-266	KLS87348	Korea, South
CC2-052	IT121464	Korea, South	CC2-267	YJ87	Korea, South
CC2-053	RAIKO	Japan	CC2-268	PI 93559	China
CC2-054	Fen dou 16	China	CC2-269	Manpung	Unknown

CC2-055	PI 95853	Korea, South	CC2-270	Blackhawk	USA
CC2-056	Daeyang	Korea, South	CC2-271	KAS524-4	Korea, South
CC2-057	Hua huang dou	China	CC2-272	Bukwang	Korea, South
CC2-058	kangwon sujib5-26	Korea, South	CC2-274	KLS714-2	Korea, South
CC2-059	KAS351-20	Korea, South	CC2-275	KLS88068-2	Korea, South
CC2-060	PI 87574	Korea, North	CC2-277	YN154	Korea, South
CC2-061	KAS638-10	Korea, South	CC2-278	KLS137-1	Korea, South
CC2-062	Qi si wa	China	CC2-279	KLS86097	Korea, South
CC2-063	YN213-2	Korea, South	CC2-280	Suwon103	Korea, South
CC2-066	PI 86904-1	Korea, South	CC2-281	KAS531-5	Korea, South
CC2-067	Sogcheong	Korea, South	CC2-282	Nezumi Meta	Korea, South
CC2-068	YJ174-2	Korea, South	CC2-283	PI 407795 A	Korea, South
CC2-069	Suigen Ao	Korea, South	CC2-284	Kantou #44	Japan
CC2-071	PI 84581	Korea, South	CC2-285	Chonggok	Korea, South
CC2-072	Huang pi feng zi wo	China	CC2-286	KLS77196-1	Korea, South
CC2-073	KLS117	Korea, South	CC2-287	IT115870	Korea, South
CC2-074	PI 88820	Korea, North	CC2-288	Giant	Korea, South
CC2-075	CS 02038	Korea, South	CC2-289	I chu tau chow	Korea, South
CC2-076	Saebyeol	Korea, South	CC2-290	IT102595	Korea, South
CC2-077	PI 399062	Korea, South	CC2-291	PI 89128	Korea, North
CC2-078	YB156	Korea, South	CC2-292	SLSB406-2	Korea, South
CC2-079	KLS88046	Korea, South	CC2-293	KLS77048	Korea, South
CC2-080	L62-667	USA	CC2-294	KLS87345	Korea, South
CC2-081	PI 556949	China	CC2-295	PI 92568	China
CC2-082	VIR 2980	Korea, North	CC2-296	PI 68484-4	China
CC2-083	IT103189	Korea, South	CC2-298	IT177322	Korea, South
CC2-084	Jeonnam jinheungwon No.11	Korea, South	CC2-300	Babmit	Korea, South
CC2-085	Baec-moc-sa-ryu	Korea, South	CC2-301	KLS85250	Korea, South
CC2-086	KLS101-2	Korea, South	CC2-302	PI 88306-1	China
CC2-087	Suwon116	Japan	CC2-303	Milyang206	Korea, South
CC2-088	Back Tac	Korea, South	CC2-304	KAS331-12	Korea, South
CC2-089	KAS183-2	Korea, South	CC2-305	PI 89143	Korea, North
CC2-090	PI 458260	Korea, South	CC2-306	Kangwon sujib3-25	Korea, South
CC2-091	KLS087062	Korea, South	CC2-307	IT177793	Korea, South
CC2-092	Bai hua chi	China	CC2-308	Kershaw	USA
CC2-094	Somyung	Korea, South	CC2-309	PI 399126	Korea, South
CC2-095	IT108810	Korea, South	CC2-311	PI 399079	Korea, South
CC2-096	PI 60269-2	Korea, South	CC2-312	PI 82555	Korea, South
CC2-097	Shillog	Korea, South	CC2-313	Yonpoong	Korea, South
CC2-098	PI 85089	Korea, South	CC2-314	Ilpumgeomjeong	Korea, South
CC2-099	Juinuni	Korea, South	CC2-315	GL2689	Korea, North
CC2-100	PI 87631-1	Japan	CC2-316	Cheonangun	Korea, South
CC2-101	PI 399089	Korea, South	CC2-317	Galmi	Korea, South
CC2-102	PI 458209	Korea, South	CC2-318	PI 62203-8	China
CC2-104	Daepung	Korea, South	CC2-319	Milyang26	Korea, South
CC2-105	GL 2624 /96	Korea, North	CC2-320	Sowon	Korea, South
CC2-106	KAS544-2	Korea, South	CC2-321	KLS123-1	Korea, South
CC2-107	Danmi2	Korea, South	CC2-322	Chungja	Korea, South
CC2-108	Doremi	Korea, South	CC2-323	PI 339982	Korea, South
CC2-110	Browngilgun	Korea, North	CC2-324	IT177388	Korea, South
CC2-111	PI 458156	Korea, South	CC2-325	PI 97150	Korea, North
CC2-112	Josaengsuri	Unknown	CC2-326	PI 340003	Korea, South
CC2-113	KLS087019	Korea, South	CC2-327	KAS574-11	Korea, South
CC2-114	KAS622-8	Korea, South	CC2-328	Kwangkyo	Korea, South

CC2-116	Ryong song	Korea, North	CC2-329	IT234975	Korea, South
CC2-117	IT104887	Korea, South	CC2-330	YJ217-2	Korea, South
CC2-118	WIR2987	Korea, North	CC2-331	YJ90	Korea, South
CC2-120	Horangi	Korea, South	CC2-332	Boone	China
CC2-121	PI 196168	Korea, South	CC2-333	IT121504	Korea, South
CC2-123	YB316-3	Korea, South	CC2-334	KAS636-21	Korea, South
CC2-124	PI 72227	China	CC2-335	KAS571-23	Korea, South
CC2-125	Soheung2	China	CC2-336	SLSJ190-2	Korea, South
CC2-126	IT208844	Korea, South	CC2-337	Baegbong	Korea, South
CC2-127	PI 475814	China	CC2-338	Namul	Korea, South
CC2-129	YN197-1	Korea, South	CC2-339	PI 407810	Korea, South
CC2-131	PI 399113	Korea, South	CC2-340	Pu Ru Ragi	Korea, North
CC2-132	Paldonamul	Korea, South	CC2-341	PI 458277	Korea, South
CC2-133	Beijing da qing don	China	CC2-342	PI 88816-S	Korea, North
CC2-134	PI 84644	Korea, South	CC2-343	OT89-06	Canada
CC2-135	PI 96549	Korea, North	CC2-344	PI 603176 A	Korea, North
CC2-136	PI 227159	Korea, South	CC2-345	Dongsan133	Japan
CC2-137	IT177337	Korea, South	CC2-346	KLS86094	Korea, South
CC2-138	IT177394	Korea, South	CC2-348	KLS85044	Korea, South
CC2-139	PI 89138	Korea, North	CC2-349	Gong xian huang dou	China
CC2-140	Cheongtae	Korea, South	CC2-350	YJ229-3	Korea, South
CC2-141	Baekmo #9	Japan	CC2-351	PI 84946-2	Korea, South
CC2-142	Yu tae	Korea, South	CC2-352	Marshall	USA
CC2-143	PI 96786	Korea, North	CC2-353	PI 84646	Korea, South
CC2-144	KAS625-19	Korea, South	CC2-354	KAS150-22	Korea, South
CC2-145	PI 96354	Korea, North	CC2-355	PI 82291	Korea, South
CC2-146	PI 159764	Korea, South	CC2-356	Myeongjunamul	Korea, South
CC2-147	PI 91073	Korea, South	CC2-357	Sugae #42	Korea, South
CC2-148	SLS B229-1	Taiwan	CC2-358	KLS606-2	Korea, South
CC2-149	KAS304-12	Korea, South	CC2-359	PI 458232	Korea, South
CC2-150	KLS 808-1	Korea, South	CC2-361	Kangwon sujib2-4	Korea, South
CC2-151	Oial	Korea, South	CC2-362	KLS87096	Korea, South
CC2-152	Si li da dou	China	CC2-363	KAS241-4	Korea, South
CC2-153	Fiskeby	Sweden	CC2-364	KAS 102-2	Korea, South
CC2-154	PI 96089-5	Korea, North	CC2-365	Suritaejung	Korea, South
CC2-155	Sugae30	Korea, South	CC2-366	YN188-3	Korea, South
CC2-156	KAS210-22	Korea, South	CC2-367	Sowon2010	Korea, South
CC2-158	Ji li huang dou	China	CC2-368	GL 2626 /96	Korea, North
CC2-159	IT177951	Korea, South	CC2-370	Danbaek	Korea, South
CC2-160	GL 2687 A	Korea, North	CC2-371	KAS502-6	Korea, South
CC2-161	KAS629-10	Korea, South	CC2-372	PI 84734	Korea, South
CC2-162	Anpyeong	Korea, South	CC2-373	Kangwon sujib5-19	Korea, South
CC2-163	YN213-1	Korea, South	CC2-374	Youngpunggeomjeong	Korea, South
CC2-164	Heihokuta	Korea, North	CC2-375	KAS 205-22	Korea, South
CC2-165	PI 54818	China	CC2-377	KLS77114-1	Korea, South
CC2-166	Xiao tie jiao	China	CC2-378	Wooram	Korea, South
CC2-167	VIR 2962	Korea, North	CC2-379	Nogwon	Korea, South
CC2-168	KAERI 590-6	Korea, South	CC2-380	IT177955	Korea, South
CC2-169	GL 2684 /95	Korea, North	CC2-381	GL 2631 /96	Korea, North
CC2-171	ORD 8139	Korea, South	CC2-382	KLS 116-1	Korea, South
CC2-172	KLS88048	Korea, South	CC2-384	PI 91725	Korea, North
CC2-173	Geomeunbak	Korea, South	CC2-385	IT177627	Korea, South
CC2-174	Xi hai zhuang da dou	China	CC2-386	YN161-3	Korea, South
CC2-175	SLS90-146	Unknown	CC2-387	PI 196175	Korea, South

CC2-176	Pureun	Korea, South	CC2-388	IT104023	Korea, South
CC2-177	KAS575-1	Korea, South	CC2-389	PI 87565	Korea, North
CC2-178	Hojang	Korea, South	CC2-390	PI 82235	Korea, South
CC2-179	Pureundogseagi	Korea, South	CC2-391	PI 83868	Korea, North
CC2-180	PI 417329	Japan	CC2-392	IT177528	Korea, South
CC2-181	KAS663-8	Korea, South	CC2-393	KLS87352	Korea, South
CC2-182	Seonbi	Korea, South	CC2-394	Jeonnam wando-2000-57	Korea, South
CC2-183	KAS 100-8-2	Korea, South	CC2-395	CS 00829	Korea, South
CC2-184	PI 83893	Korea, South	CC2-396	Soho	Korea, South
CC2-185	Nampung	Korea, South	CC2-397	IT180313	Korea, South
CC2-186	PI 171434	China	CC2-398	Hadaedu	Korea, South
CC2-187	Kangwon sujib3-24	Korea, South	CC2-399	PI 82246	Korea, South
CC2-188	KLS88066	Korea, South	CC2-400	Wonhwang	Unknown
CC2-189	PI 82544	Korea, North	CC2-401	PI 87619-1	Korea, North
CC2-190	PI 475812B	China	CC2-402	KAS640-49	Korea, South
CC2-191	IT228363	Korea, North	CC2-403	KAS641-8	Korea, South
CC2-192	PI 84646-2	Korea, South	CC2-405	IT177327	Korea, South
CC2-193	IT155162	Korea, South	CC2-406	PI 458184	Korea, South
CC2-194	Kangwon72	Korea, South	CC2-407	Keumkangdaelib	Korea, South
CC2-195	PI 83853	Korea, South	DD2-001	Bangsa	Korea, South
CC2-196	KAS505-1	Korea, South	DD2-003	K.L.S 739-2	Korea, South
CC2-197	IT177645	Korea, South	DD2-004	Cheongja3	Korea, South
CC2-198	Fu yang (30)	China	DD2-005	Daewon	Korea, South
CC2-199	Sunam	Unknown	DD2-006	PI 84669	Korea, South
CC2-200	Nonglim73	Japan	DD2-007	Daeheug	Unknown
CC2-201	PI 89154-S	Korea, North	DD2-008	Haman	Korea, South
CC2-202	KLS77170	Korea, South	DD2-009	Heugcheong	Korea, South
CC2-203	Sinpaldal	Korea, South	DD2-010	Pungwon	Korea, South
CC2-204	KLS87005	Korea, South	DD2-011	Shinhwa	Korea, South
CC2-205	PI 399108	Korea, South	DD2-012	Taekwang	Korea, South
CC2-206	Ogden	USA	DD2-013	Williams 82K	USA
CC2-207	IT102668	Korea, South	DD2-014	Yemsol	Unknown
CC2-208	PI 64698	Korea, South	DD2-015	Geomjeongol	Korea, South
CC2-209	Saedanbaek	Korea, South	DD2-016	Sinpaldal2	Korea, South
CC2-210	Savoy	USA	DD2-017	KLS105	Korea, South
CC2-211	PI 82295	Korea, South	DD2-018	Milyang12	Korea, South
CC2-212	Cheongsong sujib-1	Korea, South	DD2-019	IT104334	Korea, South
CC2-213	SLSB322-3	Korea, South	DD2-020	KAS380-14	Korea, South
CC2-214	PI 603174 A	Korea, North	DD2-021	SLSJ101-1	Korea, South
CC2-215	KAS660-12	Korea, South	DD2-022	SLSB-B-27	Korea, South
CC2-216	Shin2	Unknown	DD2-024	Peking	China

Table S5: Primers used in this study.

Forward primer	Reverse primer	Tm(℃)	Purpose	Target (region / variant / Gene)
AGGACCATGAAACAGGGTTG	CCAGCAAGTGGTGAAGGATT	60	SSR genotyping	*qBR6-1*
ACACACCCAAACCCAACATT	GATTTTGCGGTGGAGGTAGA	60		*qBR6-1*
CTTGGCACCTGCAGATTCTT	CCTCTCACAATCGCAATGAC	67	SNP genotyping	Gm06:20868970_C/T
CTACAGATTGAGAAAGACAGCACAA	CTGCAATCAGATTTGCTACAACTAA	60		Gm06:20870179_C/G, Gm06:20870246_A/T, Gm06:20870348_G/T, Gm06:20870655_C/A
TCAAAATCGAATCAAACCGA	TTTTCAATCCAACCCAACTCA	60		Gm06:20870775_C/T, Gm06:20870777_T/C, Gm06:20871174_G/A
GTGCAAAGTGGAGCAACAGA	AGATGCTGCTTGCCTATGCT	60	qRT-PCR	Glyma.06G209000 (NF-YB13 nuclear factor Y, subunit B13)
AGAGCTGGCTACGCCAATAA	CACACTCTCTTCTGGTGGCA	60		Glyma.06G209100 (MIZU-KUSSEI like protein, *MIZ1*)
CCCATGCTATTTTGCAGGTT	GATGGGCTGTTGGCAGTAGT	60		Glyma.06G209200 (ATP binding microtubule motor family protein)
CTGGAAGGAGTTCTGCAAGG	CAATCCCCGACAGTTTTGTT	60		Glyma.06G209300 (homeodomain GLABROUS 2, *HDG2*)
ACCGAGCAGAAACAAGAGGA	GTGGAAGCTCATGTCTGCAA	60		Glyma.06G209400 (P-loop containing nucleoside triphosphate hydrolases superfamily protein)
CTCGTACCCCTCCACAAAAA	ACGTGTGGCAGGTGAACATA	60		Glyma.06G209600 (Adenine nucleotide alpha hydrolases-like superfamily protein)
CTCCTCCACGAATGCAATTT	AACGCATTCCAACCTTGAAC	60		Glyma.06G209700 (NagB/RpiA/CoA transferase-like superfamily protein)
CACAGAATCTGCCAACAGGA	AGCAATGGCTGATTGCTCTT	60		Glyma.06G209800 (Hydroxyproline-rich glycoprotein family protein, *CHUP1*)
GCCGTTCTCTTTCTCGTCAC	GGTCTTTGACTCGGTTCAGC	60		Glyma.06G209900 (Oleosin family protein)
GTGAGGGAGCAGTGGAGGAT	TAAATGACGCCCGAGAAATC	61		Glyma.06G210200 (not annotated)
AATCAGTGCATTTGACCCTCTT	AGCTAGCACTCCACGATTTCTC	57		Glyma.06G210600 (TCP transcription factor, *BRC1*)
CAAGTTGCACACGCACTCTT	TAATCGCAATGCATCCAGAG	59		Glyma.06G210400 (TFIIE alpha subunit)
CGGTGGTTCTATCTTGGCATC	GTCTTTCGCTTCAATAACCCTA	60		*ACTIN11* (reference gene, *ACT11*)

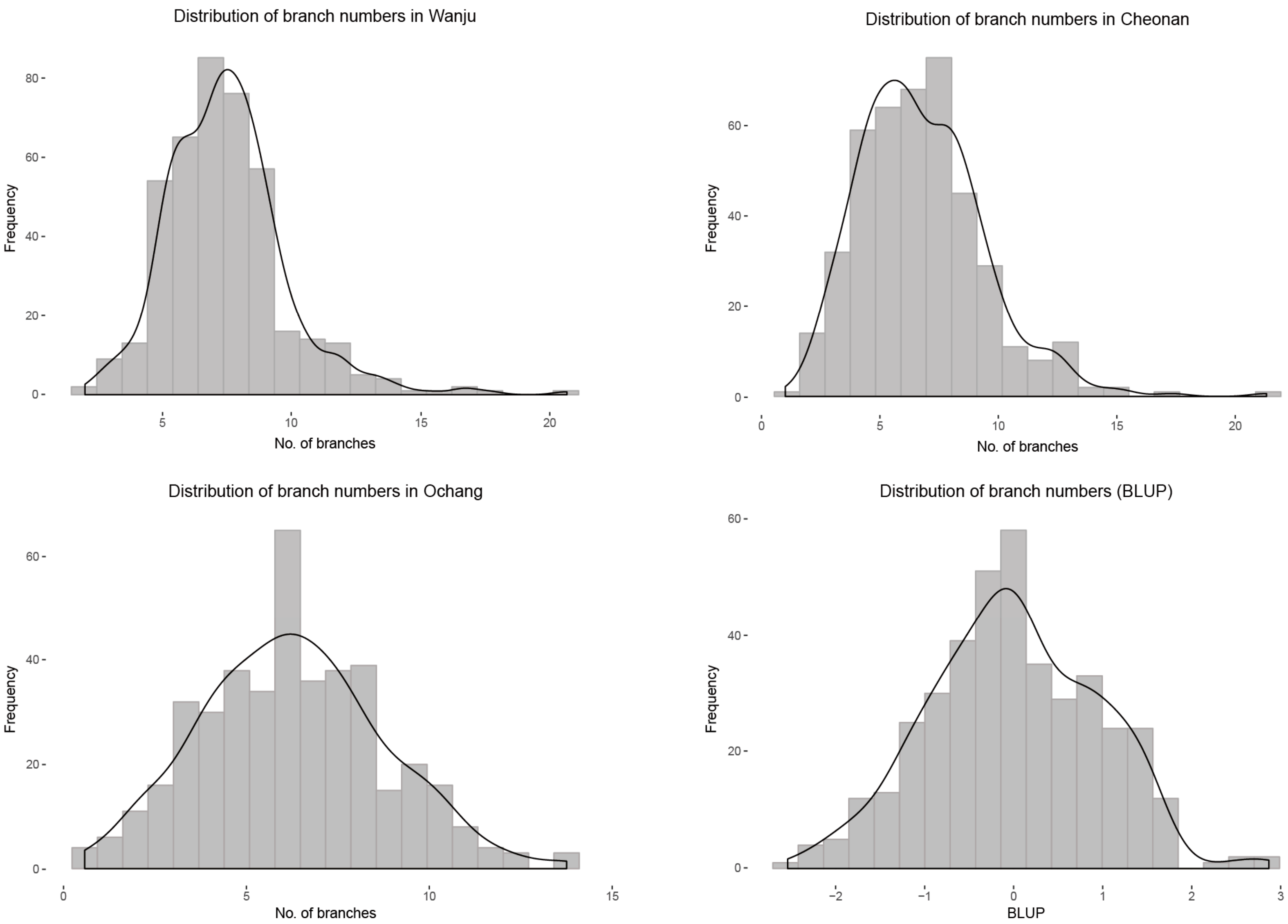

Figure S1. Phenotypic distribution of branch numbers in the soybean core collection according to three different locations of cultivation (Wanju, Cheonan, and Ochang) and best linear unbiased predictor (BLUP) values. Black lines represent the density curves of phenotypes.

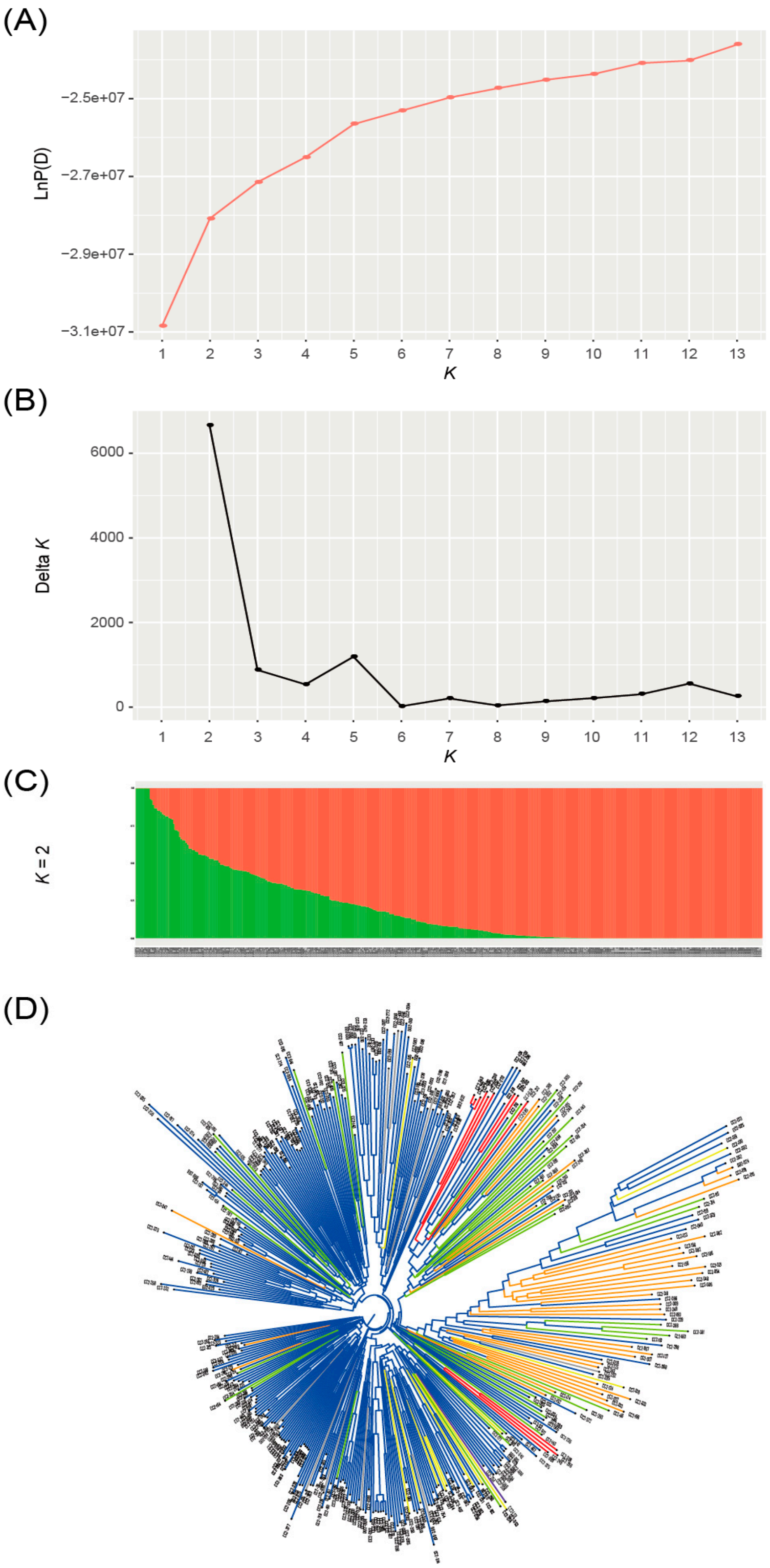

Figure S2. Population structure and phylogenetic tree of 400 soybean accessions. (A,B) LnP(D) and *ΔK* based STRUCTURE analysis. (C) Population structure classified into two subgroups under optimal *K*=2. (D) A neighbor-joining tree of the 400 soybean accessions used in GWAS.

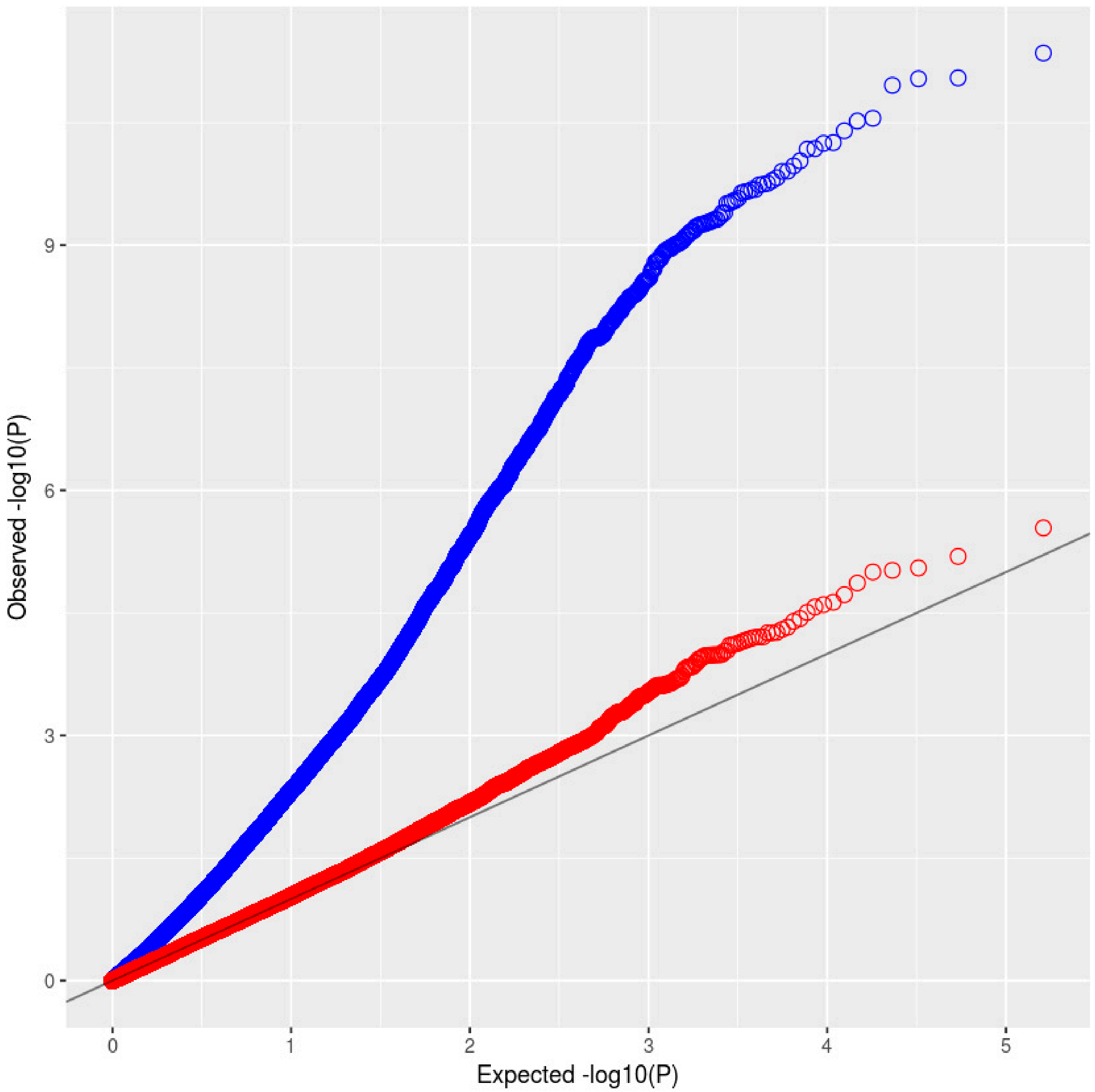

Figure S3. Quantile-quantile (QQ) plots of two genome-wide association study (GWAS) models for branch numbers. Blue and red dots represent QQ-plot of GLM +Q and MLM+Q,K models, respectively.

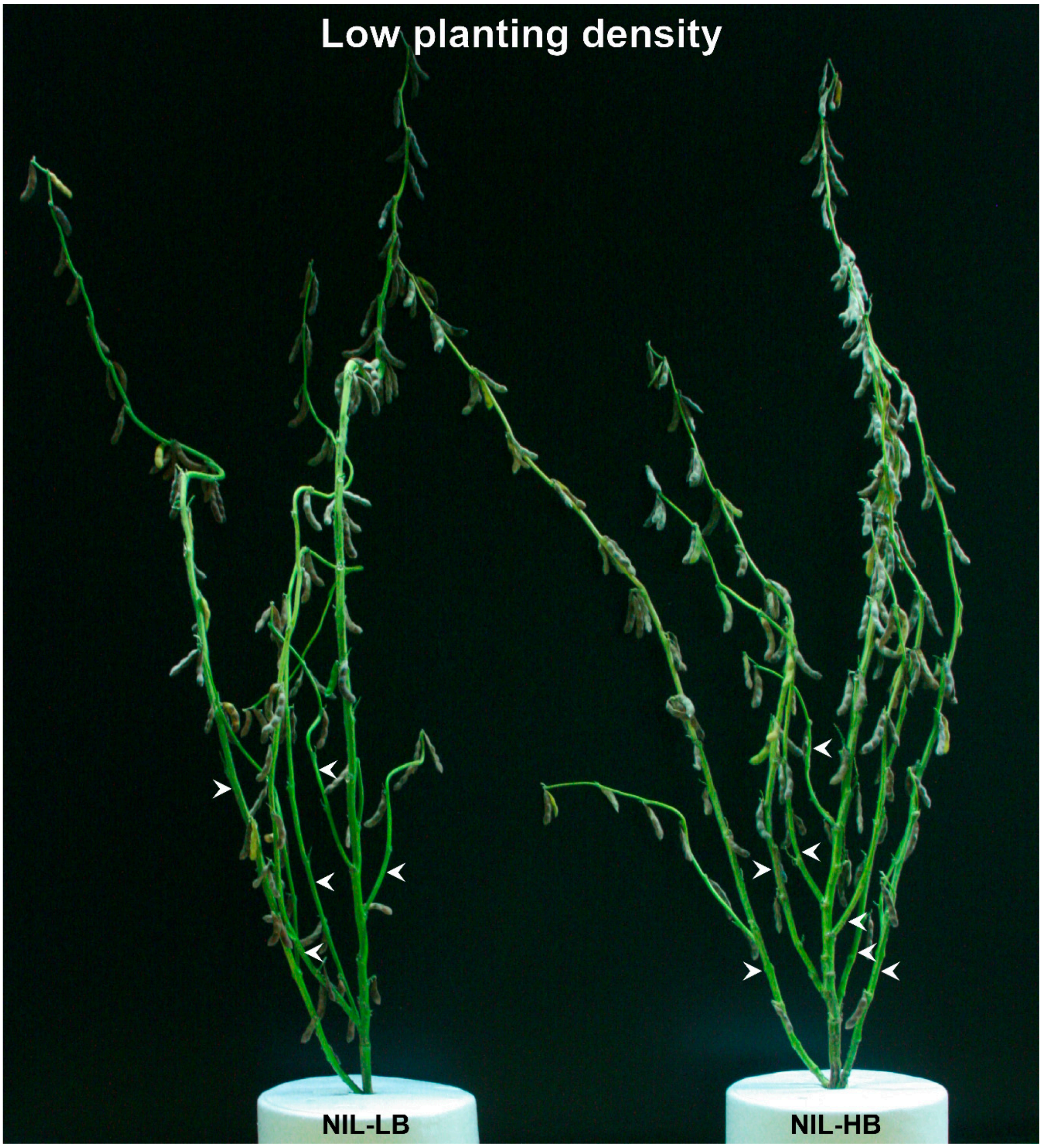

Figure S4: NIL-LB and NIL-HB cultivated under the low planting density condition. White arrows indicate branches outgrowing from the main stem.
